# Job satisfaction among pharmacy professionals working in public hospitals and its associated factors, eastern Ethiopia

**DOI:** 10.1186/s40545-020-00209-3

**Published:** 2020-05-11

**Authors:** Yohanes Ayele, Behailu Hawulte, Tilayie Feto, G. Vijai Basker, Yadeta Dessie Bacha

**Affiliations:** 1 Department of Clinical Pharmacy, School of pharmacy, College of Health and Medical Sciences, Haramaya University, Harar, Ethiopia; 2 School of public health, College of Health and Medical Sciences, Haramaya University, Harar, Ethiopia; 3 School of nursing and midwifery, College of Health and Medical Sciences, Haramaya University, Harar, Ethiopia

**Keywords:** Pharmacy professionals, Job satisfaction, Eastern Ethiopia

## Abstract

**Background:**

Poor job satisfaction has been associated with less productivity and high staff turnover. Various factors are thought to contribute for job dissatisfaction among pharmacy professionals and very limited studies have been conducted in eastern part of Ethiopia. Therefore, the current study was aimed to assess the level of job satisfaction among pharmacy professionals and its predictors.

**Methods:**

A cross-sectional study was conducted among 232 pharmacy professionals to assess level of job satisfaction in public hospitals located in the eastern Ethiopia. The data were collected using self- administered semi-structured questionnaires. Data were entered into Epi-Data version 3.1 and exported to STATA version 14.2 for analysis. Associations between the dependent and independent variables were assessed by multivariate analysis using an Adjusted Odds Ratio (AOR) at a 95% confidence interval (CI) and *p*-value less than 0.05 was considered as significant.

**Results:**

A total 220 questionnaires were found complete and included in the analysis. The mean age of participants was 27.6(SD + 4.1). More than half of the respondents (55.4%) had a bachelor degree and the majority (86.4%) were working less than 40 h per week, mostly in dispensing units (75.4%). About one third of the participants (32.7, 95% CI; 26.8–39.2) were found to be satisfied with their job. Age category of 20 to 25 years in reference to age greater than 30 years (AOR = 3.5, 95% CI; 1.1–9.7)**,** holding a bachelor degree in reference to having diploma (AOR = 4.2, 95% CI; 1.8–10.00), working for more than 40 h per week (AOR = 6.2, 95% CI, 2.4–16), and working in dispensing units (AOR = 2.4, 95% CI; 1.1–5.5) were found to have strong association with job dissatisfaction.

**Conclusion:**

In this study, the job satisfaction levels of pharmacy professionals were found to be very low. The age category of 20 to 25, holding a bachelor degree, working for more than 40 h per week, and working in dispensing unit were found to be strong predictors of job dissatisfaction. Hence, pharmacy directors and hospital administrators should work to reduce unnecessary workload on the staffs and create good working climate.

## Introduction

The services delivered by the hospital pharmacy unit are a vital component of an institutional health care system. Medication dispensing and drug distribution, compounding, medication utilization review, adverse drug reaction monitoring, drug information service, participating in drug and therapeutics committee are some of the services delivered by this unit [1]. Furthermore, in the past few decades, there has been a major shift in paradigm of pharmacy practice embracing the concept of clinical pharmacy which is intended to increase the involvement of pharmacy personnel in direct patient care [2, 3].

According to Avedis Donabedian, the health care quality can be measured through measuring the structure, process and outcome of health care system [4]. From the process aspect, one of the quality service indicators that have been widely used is the level of professionals’ job satisfaction. By the same token, determining the job satisfaction level of the pharmacy professionals is expected to give insight in to the quality of pharmaceutical service [5, 6].

The key component of appropriate human resource management is keeping workers motivated which is highly dependent on their level of satisfaction. Job satisfaction is not only related to productivity but also the overall quality of life of the professionals [7]. Studies have demonstrated the importance of job satisfaction to an organization in terms of its positive relationship with individual performance, productivity, employee relations, physical and mental health and life satisfaction [8, 9]. On the other hand, poor job satisfaction has been associated with less productivity and high staff turnover [10–12].

Various factors are reported to affect job satisfaction of professionals which generally are grouped as intrinsic and extrinsic factors. The intrinsic factor included achievement, recognition and responsibility whereas extrinsic factors are interpersonal relations, working environment, salary among others [13–15]. Similarly, job satisfaction of pharmacists is reported to be affected by many factors including work environment and professional interaction, salary, governmental policies, promotion opportunities [8, 16–20]. Hence, identifying the influencing factors and maintaining job satisfaction in the work place will have a significant effect on staff retention and providing appropriate patient care.

In Ethiopia, hospital pharmacy professionals are a key component of the health care system. The Pharmacy professionals in these settings are expected to provide dispensing services in four departments; outpatient, inpatient department, antiretroviral therapy, and emergency unit. The inventory and logistics management, and clinical pharmacy services which is recently introduced model of pharmacy practice is another practice area for Ethiopian hospital pharmacists [21]. Regardless of the much-needed service of these professionals, the country suffers from serious pharmacy workforce scarcity, a study reporting pharmacist density of 2.38 per 100,000 populations which is the smallest number compared to other African countries [22].

Moreover, in Ethiopia, there is growing need for pharmacy professionals as the health care institutions continue to expand and the government looks for full implementation of clinical pharmacy service [21, 23]. To make things worse, reports indicate poor job satisfaction among pharmacy professionals amid inadequate compensation, inadequate management systems, heavy workloads, and lack of technical support among others [22, 24].

As far as our knowledge goes, there are no sufficient and effective studies highlighting the pharmacy professionals’ job satisfaction level in public hospitals, particularly in the eastern part of the country. We believe that measuring the level of job satisfaction of pharmacy professionals would be important as it can generate information that could help employers and organizations to change their approach in the management of pharmacy professionals. Therefore, the aim of this study was to assess the level of job satisfaction and associated factors among pharmacy professionals in Eastern part of Ethiopia.

## Methods

### Study setting and period

A cross-sectional study was conducted at public hospitals in Eastern Ethiopia from September to December, 2018 to assess pharmacy professionals’ job satisfaction and associated factors. Three regional states are located in eastern part of Ethiopia; Harar, part of Oromia, and Ethiopian Somali region. This study was conducted in western and eastern Hararghe zone of Oromia region, Harar region, Diredawa administrative city and Fafan Zone of the Ethiopian Somali region. In the study area, there were 13 public hospitals; two specialized hospitals, eight general hospitals, and three primary hospitals.

### Study population

All health care professionals working in study hospitals were the source population. All pharmacy professionals working in the study hospitals during the study period were included in the study. Newly recruited pharmacy professionals who worked in the respective hospitals for less than 3 months were excluded from the study.

### Sample size and sampling technique

The number of pharmacy professionals participated in the job satisfaction survey was determined using the single proportion formula. The sample size was determined assuming 60.8% prevalence of pharmacy professional job satisfaction [25], sampling error of 5 and 95% confidence interval. Hence, the sample size calculated was 366. Since the total (source) population in the current study were less than 10,000, we used the population correction formula [26].

$$ nf=\frac{n}{1+\left(\frac{n}{N}\right)} $$

Where nf = final sample, N = total number of pharmacy professionals working in the hospitals included in the study during data collection period which was equal to 500 according to report from hospitals administration.

Accordingly, a total of 232 sample size was considered after adding 10% non-response rate. The participants included in the study were sampled from all 12 hospitals. The final sample was distributed throughout the study hospitals proportionally considering the number of pharmacy professionals working in each hospital during the data collection period to ensure the representativeness of the sample.

### Data collection tools and procedures

A self-administered semi-structured questionnaire was used to assess the level of pharmacy professionals’ job satisfaction. The questionnaire was adapted from previous similar study [27]. The tool contained three sections; socio-demographic characteristics, pharmacy professionals’ characteristics, and the level of pharmacy professional job satisfaction. The later section had three categories of questions: items on working environment designed to assess satisfaction with physical working environment comprising 11 questions; items on professional interactions designed to assess inter-professional interaction with fellow pharmacy professionals, nurses and physicians comprising of seven questions and items on incentive and recognition designed to asses satisfaction with monetary compensation and professional recognition comprising of seven questions. A five-point Likert scale, value ranging from 1 (strongly disagree) to 5 (strongly agree), was used to rate the questions. Pre-test was conducted and necessary modification was done. The content of questions was modified specifically vague and complex statements were corrected to improve understandability. Additionally, instruction for respondents was revised based on the pre-test finding.

### Data processing and analysis

The coded data were entered into Epi-Data version 3.1 after checking for completeness and exported to STATA version 14.2 for analysis. Data were described using proportion, mean and standard deviation. After checking for normality of data, we used mean score to determine magnitude of overall job satisfaction [28, 29]. Consequently, respondents with an average score of less than mean value, were classified as dissatisfied, and those with an average score of mean value and above were considered as satisfied. Bivariate and multivariable analyses were done to identify the factors associated with providers’ level of satisfaction. The variables with a *p*-value of less than 0.25 in the bivariate analysis were included in the multivariable analysis to account for all important variables [30, 31]. Associations between the dependent and independent variables were assessed using adjusted odds ratio (AOR) at a 95% confidence interval (CI) and the *p*-value less than 0.05 was considered as significant.

## Results

### Socio-demographic characteristics of participants

A total of 232 questionnaires were distributed and overall 220 participants filled the questionnaires completely and therefore used for the data analysis. As can be seen from Table 1, considerable portion (60.9%) of the participants were male. Considering the age of professionals, the mean age of the participants was 27.6(SD + 4.1). More than half of the professionals (59.1%) were single. Regarding monthly income, exactly half of the participants were receiving the salary of ranging from 2501 to 5000 Ethiopian birr (ETB) and most of the participants had no other source of income.

**Table 1:** Socio-demographic characteristics of pharmacy professional assessed for job satisfaction in public hospitals in Eastern Ethiopia, 2017 (*n* = 220)

Variables	Number (%)
**Sex**	
Male	134 (60.9)
Female	86 (39.1)
**Age**	
20–25	78 (35.4)
26–30	108 (49.1)
above 30	34 (15.5)
**Marital status**	
Single	130 (59.1)
Married	86 (39.1)
Divorced	4 (1.8)
**Current religion**	
Muslim	86 (39.1)
Orthodox	94 (42.7)
Protestant	24 (10.9)
Catholic	2 (0.9)
Others	14 (6.4)
**Monthly salary (**ETB)^a^	
Less than 2500	26 (11.8)
2501–5000	110 (50.0)
> 5000	84 (38.2)
**Other source of income**	
Yes	20 (9.1)
No	200 (90.9)

^**a**^ETB-Ethiopian Birr, based on the Ethiopian civil servant monthly salary scale

### Pharmacy professionals’ characteristics

As Table 2 shows, more than half of the respondents (55.4%) had a bachelor degree in pharmacy during the data collection period while the remaining held college diploma. The average (±SD) service years of providers was 3.7 (±2.9) years and the average (±SD) working hours of the providers was 41.1 (±5.2) hours per week. As to the professionals’ current working unit, a large proportion of participants (75.4%) were working in different dispensing unit of hospitals. It was noted that only 12.7% professionals were working in the ward to deliver clinical pharmacy services. Regarding technical support or supervision participants received during previous one-year period, slightly more than half of the participants (56.4%) reported lack of technical support or supervision. With relation to the professionals’ opinion on the measure required to increase job satisfaction, the participants mentioned the need for more knowledge or updates or training 158 (71.8%), more incentives 114 (51.8%), and better facility infrastructure 106 (48.2%).

**Table 2:** Pharmacy professionals’ characteristics assessed for job satisfaction in public hospitals in Eastern Ethiopia, 2018 (*n* = 220)

Variables	Number (%)
**Qualification**	
Diploma in pharmacy	98 (44.6)
Degree in pharmacy	122 (55.4)
**Service year/s**	
< 2	60 (27.3)
2–4	88 (40.0)
> 4	72 (32.7)
**Working hours per week**^**a**^	
40 h and less	190 (86.4)
more than 40 h	30 (13.6)
**Current working unit**	
Dispensing	166 (75.4)
Inpatient Ward	28 (12.7)
Inventory and drug supply control	10 (4.6)
Administrative activities	16 (7.3)
**Dispensing unit (*n*** **= 166)**	
Outpatient unit	94 (56.6)
Inpatient unit	40 (24.1)
Emergency Drugs unit	12 (7.2)
Antiretroviral Drugs unity	20 (12.1)
**Technical support or supervision**	
No	124 (56.4)
Yes	96 (43.6)
**Measures to increase job satisfaction**^**b**^	
More knowledge or updates or training	158 (71.8)
More incentives for professionals	114 (51.8)
Better facility infrastructure	106 (48.2)
Less workload	90 (40.9)
More support from supervisor	70 (32.4)
More quality supplies or stock	66 (30.3)
More autonomy or independence	38 (17.3)

^**a**^Professionals are expected to serve 40 h per week (five working days), ^**b**^the sum does not give 100%

### Job satisfaction among pharmacy professionals

**Fig. 1:**
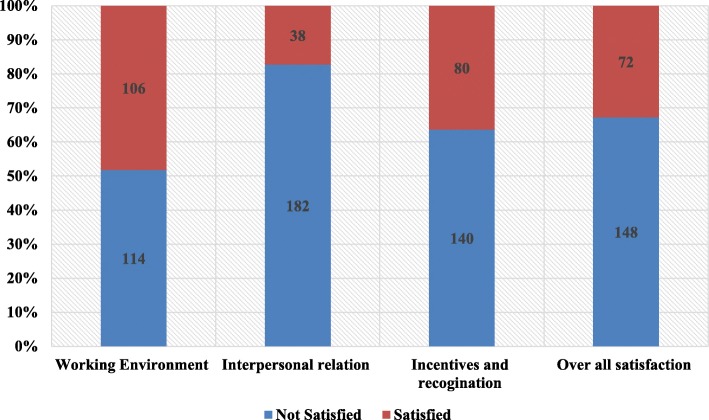
Level of overall job satisfaction and satisfaction on working environment, interpersonal relations, and incentive and recognition, among Pharmacy professionals in Easter Ethiopia, 2018 (*n* = 220)

The job satisfactions of the professionals were assessed using 25 questions which were grouped in to three categories; working environment, interpersonal interactions, and incentive and recognition. We calculated both the overall satisfaction level and satisfaction level within each category. Table 3 shows response of all participants for each question. Figure 1 depicts the job satisfaction level of the participants summarized in three categories. In this study, only about one third of the participants (32.7, 95% CI; 26.8–39.2) were found to be satisfied with their job. The mean (±SD) satisfaction level calculated out 5 was 2.8(+ 0.7).

**Table 3:** Proportions of pharmacy professionals’ response to job satisfaction questions in Easter Ethiopia, 2018 (*n* = 220)

Questions	Strongly disagree n (%)	Disagree n (%)	Neutral n (%)	Agree n (%)	Strongly agree n (%)
**Working environment**					
The opportunity for promotion in this hospital is good.	20 (9.1)	52 (23.6)	58 (26.4)	74 (33.6)	16 (7.3)
I am proud to be working for this hospital.	16 (7.3)	30 (13.6)	34 (15.5)	112 (50.9)	28 (12.7)
Rules and regulation in this hospital are applied equally.	32 (14.6)	38 (17.3)	78 (35.5)	56 (25.5)	6 (7.3)
My supervisor gives serious consideration to employee complaints.	20 (9.1)	34 (15.4)	80 (36.4)	78 (35.5)	8 (3.6)
Employees have a sufficient amount of freedom to decide how they do their work.	22 (10.0)	38 (17.3)	74 (33.6)	74 (33.6)	12 (5.5)
Staffing is adequate; enough employees are hired to cover the workload in the pharmacy	32 (14.6)	80 (36.4)	36 (16.4)	54 (24.5)	18 (8.1)
My supervisor is capable of providing proper guidance.	24 (10.9)	36 (16.4)	80 (36.4)	74 (33.6)	6 (2.7)
The hospital management respects and treats pharmacy professionals similar to other health professionals in the hospital.	22 (10.0)	56 (25.5)	46 (20.9)	72 (32.7)	24 (10.9)
Scheduling of work hours takes into account individual employee needs.	16 (7.3)	44 (20.0)	68 (30.9)	80 (36.4)	12 (5.4)
I am comfortable with my work load.	34 (15.4)	74 (33.6)	38 (17.3)	58 (26.4)	16 (7.3)
There is suitable working environment (lighting, air condition, toilet facilities, and rest room)	56 (25.5)	58 (26.4)	44 (20.0)	50 (22.7)	12 (5.4)
**Professional interaction**					
Physicians consult me on professional matters.	16 (7.3)	22 (10.0)	44 (20.0)	92 (41.8)	46 (20.9)
Physicians cooperate when I communicate professional matters with them.	14 (6.4)	20 (9.1)	42 (19.1)	120 (54.5)	24 (10.9)
My fellow employee pharmacy professionals treat me with professional respect.	14 (6.3)	16 (7.3)	42 (19.1)	90 (40.9)	58 (26.4)
My fellow employees are friendly.	6 (2.8)	28 (12.8)	24 (11.0)	94 (43.1)	66 (30.3)
Nurses cooperate when I communicate professional matters with them.	8 (3.6)	14 (6.4)	38 (17.4)	130 (59.0)	30 (13.6)
Nurses often initiate consultations with me on professional matters.	10 (4.6)	28 (12.7)	46 (20.9)	112 (50.9)	24 (10.9)
I am satisfied with the working relationships I have with other staffs.	18 (8.2)	16 (7.2)	32 (14.6)	102 (46.4)	52 (23.6)
**Incentive and recognition**					
The monetary rewards I receive from my work are appropriate.	52 (23.8)	52 (23.9)	58 (26.6)	54 (24.8)	2 (0.9)
My salary is fair considering the service I give.	52 (23.6)	78 (35.5)	34 (15.5)	50 (22.7)	6 (2.7)
I receive adequate incentive for my wok.	60 (27.3)	70 (31.8)	30 (13.6)	42 (19.1)	18 (8.2)
My talents are fully utilized on my job.	34 (15.5)	26 (11.8)	76 (34.5)	70 (31.8)	14 (6.4)
I am willing to continue the current job in future too.	24 (10.9)	26 (11.8)	36 (16.4)	96 (43.6)	38 (17.3)
I often leave work with a feeling that I’m doing something which I enjoy.	16 (7.3)	22 (10.0)	40 (18.2)	122 (55.4)	20 (9.1)
Knowing what I know now, if I had to decide all over again, I would still choose pharmacy as my profession.	18 (8.2)	22 (10.0)	30 (13.6)	96 (43.6)	54 (24.6)

In regard to satisfaction level of in each category, just under one half of the participants (48.2%) were satisfied with the physical working environment. The figure drops further to less than one fifth (17.3%) for professional interaction. Although it was not comparable with satisfaction seen on the professional interaction, the satisfaction level seen among professionals on the incentive and recognition related issues was low, only one third (36.4%) of the respondents reported satisfaction.

### Determinants of job satisfaction among pharmacy professionals

In this study, age, qualification level, working hours and current working unit of participants were found to be strong predictors of job satisfaction for pharmacy professionals. In relation to age, participants who were in the age range of 20–25 years were found to be 3.5 times (AOR = 3.5, 95% CI; 1.1–9.7) more likely to be dissatisfied compared with professionals who were above 30 years.

Another determinant of job satisfaction seen in this study was level of qualification of participants. According to our finding, pharmacy professionals who held a bachelor degree were found to be 4.2 times (AOR = 4.2, 95% CI;1.8–10.00) more dissatisfied in reference to diploma holders. In the present study, working hours per week were also found to have significant association with job satisfaction of participants. As it is shown in Table 4, respondents who were working for more than 40 h per week were 6.2 times (AOR = 6.2, 95% CI; 2.4–16) more dissatisfied in reference with participants who were engaged 40 h or less per week. Finally, working in dispensing units was found to be a source of dissatisfaction. It was found that pharmacy professionals working in dispensing units were 2.4 times (AOR = 2.4, 95% CI; 1.1–5.5) dissatisfied in reference to respondents who were working in other units.

**Table 4:** Determinants of job satisfaction among pharmacy professionals in Easter Ethiopia, 2018 (*n* = 220)

Variables		Satisfaction		COR (95%CI)	AOR (95% CI)	***P*** value
		No (***n*** = 148) n (%)	Yes (***n*** = 72) n (%)			
Sex	Female	58 (39.2)	28 (38.9)	1	1	0.741
	Male	90 (60.8)	44 (61.1)	1.01 (0.6–1.8)	0.9 (0.4–1.7)	
Age	> 30	26 (17.6)	8 (11.1)	1	1	
	26–30	78 (52.7)	30 (41.7)	1.3 (0.5–3.1)	1.3 (0.5–3.3)	0.736
	20–25	44 (29.7)	34 (47.2)	2.5 (1.01–6.2)	**3.5 (1.1–9.7)**	**0.041**
Monthly salary	< 2500	21 (14.2)	5 (7.0)	1	1	
	2500–5000	68 (45.9)	42 (58.3)	2.6 (0.9–7.4)	1.6 (0.5–5.1)	0.375
	> 5000	59 (39.9)	25 (34.7)	1.8 (0.6–5.2)	0.9 (0.2–3.4)	0.885
Qualification	Diploma	72 (48.7)	26 (36.1)	1	1	**0.002**
	Degree	76 (51.3)	46 (63.9)	1.7 (0.9–3.0)	**4.2 (1.8–10.0)**	
Service years	< 2	36 (24.3)	24 (33.3)	1	1	
	2–4	62 (41.9)	26 (36.1)	0.6 (0.3–1.3)	1.4 (0.6–3.3)	0.430
	> 4	50 (33.8)	22 (30.6)	0.7 (0.3–1.4)	1.1 (0.4–3.2)	0.690
Working hours per week	≤40 h	136 (91.9)	54 (75.0)	1	1	**0.000**
	> 40 h	12 (8.1)	18 (25.0)	3.8 (1.7–8.4)	**6.2 (2.4–16.1)**	
Current working unit	Non-dispensing units ^**a**^	40 (27.0)	14 (19.3)	1	1	**0.039**
	Dispensing units	108 (73.0)	58 (80.6)	1.5 (0.8–3.1)	**2.4 (1.1–5.5)**	
Technical support or supervision	No	82 (55.4)	42 (58.3)	1	1	0.970
	Yes	66 (44.6)	30 (41.7)	0.9 (0.5–1.6)	0.9 (0.5–1.9)	

Variables with significant association is presented in bold, ^**a**^inventory and drug supply control, administrative activities and inpatient ward, COR-Crude Odds Ratio

## Discussion

This study was conducted to assess overall job satisfaction and its determinants among pharmacy professionals working in public hospitals in eastern Ethiopia. In our study only 32.7% of the professional were satisfied with their job. The mean satisfaction level of was 2.8(+ 0.7). Factors such as being at age 20 to 25 years, level of qualification, working hours per week, and current working unit were strongly associated with respondents’ job satisfaction.

It is obvious that hospital pharmacy services suffer from lack of quality in Ethiopia [24, 32]. Job satisfaction is a key factor affecting professional motivation and productivity since satisfied workers are in better position to deliver the service to the expected standard. In the present study, the job satisfactions of pharmacy professionals working in public hospitals were found to be very low, only about one in three of participants reporting satisfaction (32.7, 95% CI; 26.8–39.2), the mean satisfaction 2.8(+ 0.7) measured out of five. This finding is consistent with research conducted in Gondar university hospital in which the mean satisfaction was 2.7 [24], and report from Addis Ababa, 3.0 ± 1.1 (mean ± SD) [22]. However, the finding is lower than Australian study 3.6 +/− 0.7 [29], and a report by Mohamed mansor manan et al., 52% [33]. It is also significantly lower than a study conducted to assess job satisfaction among health care professionals in the university hospital of Gondar (54%) [34] and Jimma, Ethiopia (41.4%) [35]. Consequently, it is very important for the hospital management to look deep in to the case and take appropriate measures.

Studies have identified the number of determinants for job satisfaction, including demographic characteristics such as age, gender, monthly salary, education among others though the results were inconclusive [36–39]. One of the demographic variables often reported to have an association to job satisfaction was age. Age in general is reported to have U-shaped association with job satisfaction [16], the professionals having good satisfaction in the beginning of their career which drops in their mid, only to get better at the end of their career. There are also sizeable reports indicating better satisfaction in both ends of professionals’ career [33, 40–42].

In the present study, participants who were between the ages of 20 to 25 were more dissatisfied with their job compared to older fellow professionals (AOR = 3.5, 95% CI; 1.1–9.7). This finding is in agreement with study conducted in Malaysia which reported better satisfaction in the age group of 35–45 years compared to younger pharmacists [33] and elsewhere [22, 36, 40, 41, 43, 44]. The possible explanation for this finding is a high expectation of new employee and unmet need leading to dissatisfaction [41, 43]. It is also good to consider the methodological variation and the effect of confounding variables while comparing these results.

Generally, studies assessed the effect of qualification level on one’s job satisfaction is indecisive. There is strong evidence implying that people with higher level of education have better levels of job satisfaction because of better opportunities that come along [45]. This might stand true for pharmacy professional as well particularly for those who achieve higher education than Bachelor degree. In addition, sense of achievement through attaining higher degree is also thought to be a possible source of satisfaction [18, 42, 43, 46]. In Ethiopia, three ladders of education level are expected for pharmacy professionals working in hospital pharmacy; college certificate or diploma, Bachelor of pharmacy and masters of Science in pharmacy. Coincidentally, all the pharmacy professionals who participated in this study had either diploma or degree. In the present study, pharmacists with degree qualification were more likely to be dissatisfied compared to professionals having diploma (AOR = 4.2, 95% CI; 1.8–10.0). Decreased satisfaction seen in this setting might be due to perceived lack of their skill being fully utilized [36, 47–49].

Work load can be an important source of job dissatisfaction. In our study, we found professionals who were working for more than 40 h per week which is minimum hours civil servants are expected to serve per week in Ethiopia dissatisfied with their job. This is in line with other studies which reported long working hours as a source of discontent among pharmacy professionals [16, 50–52]. Hence, management of hospital particularly hospital pharmacy managers should distribute the activities in a way it does not create workload on the employee. Furthermore, management should ensure presence of adequate human resource to balance the work load. This can be achieved through recruiting new staffs and increasing efficiency and expanding pharmacy workforce at national level.

In Ethiopian health care system, pharmacy professionals play multiple role including working in ward, dispensing, inventory and logistics management and administration. In the present study, pharmacy professionals working in dispensing units were found to have less satisfaction level compared to professionals working in other units (AOR = 2.4, 95% CI; 1.1–5.5). There are studies indicating pharmacists’ preference of clinical activities which thought to be more challenging over distributive functions [36, 41, 53]. However, given that the practice of clinical pharmacy is in its infancy stage in Ethiopia, the possible explanation to high dissatisfaction among pharmacy professionals working in dispensing units could be high work load and the less comfortable working environment [32, 34, 35, 54].

This research is not without limitation. Firstly, since it was cross-sectional study, we were not able to establish cause-effect relationship between factors that were reported to have strong association with job satisfaction. Secondly, this research was limited to pharmacy professionals working in public hospitals; hence it should be generalized cautiously. What is more it was assumed that the job satisfaction seen is due to job related activities while some personal factors such as socio-economic factors might have affected the reported satisfaction level. Finally, the data is also subjected to bias as it is self-reported.

## Conclusion

In this study, the job satisfaction levels of pharmacy professionals were found to be very low. Regarding factors affecting job satisfaction, different factors were found to have association. From sociodemographic variables, age (category of 20 to 25), was found to be strong predictors of job dissatisfaction however, gender and other variables didn’t have impact on job satisfaction. The professionals’ qualification level and working condition were also found to have influence on job satisfaction; holding a bachelor degree, working for more than 40 h per week, and working in dispensing unit showing strong association with job dissatisfaction. Hence, pharmacy directors and hospital administrators should work to reduce unnecessary workload on the staffs and make the working environment more comfortable.

## Data Availability

The data collection tools are attached as an additional supporting file. The datasets are available from the corresponding author on reasonable request.

## Abbreviations

AOR: Adjusted odds ratio
CI: Confidence interval
SD: Standard Deviation
